# Assessing exposure to weight stigma: development and initial validation of the Weight Stigma Exposure Inventory (WeSEI)

**DOI:** 10.1186/s40337-024-01168-9

**Published:** 2025-01-06

**Authors:** Kamolthip Ruckwongpatr, I-Hua Chen, Iqbal Pramukti, Po-Ching Huang, Janet D. Latner, Kerry S. O’Brien, Xuelian Wang, Jung-Sheng Chen, Servet Üztemur, Chien-Chin Lin, Yen-Ling Chang, Wei-Leng Chin, Mark D. Griffiths, Chung-Ying Lin

**Affiliations:** 1 Institute of Allied Health Sciences, College of Medicine, National Cheng Kung University, Tainan, 701401 Taiwan; 2 Qufu Normal University, Chinese Academy of Education Big Data, Qufu, 273165 China; 3 Faculty of Nursing, Universitas Padjadjaran, Bandung, West Java 45363 Indonesia; 4 School of Physical Therapy, Graduate Institute of Rehabilitation Science, College of Medicine, Chang Gung University, Taoyuan, 333323 Taiwan; 5 Department of Psychology, University of Hawaii at Manoa, Honolulu, HI 96822 USA; 6 Faculty of Arts, School of Social Sciences, Monash University, Melbourne, VIC 3800 Australia; 7 Yancheng Mechatronic Branch of Jiangsu Union Technical Institute, Yancheng, 224006 China; 8 Department of Medical Research, E-Da Hospital, I-Shou University, Kaohsiung, 824005 Taiwan; 9 Department of Turkish and Social Sciences Education, Faculty of Education, Anadolu University, 26470 Eskişehir, Türkiye; 10 Department of Laboratory Medicine, National Taiwan University Hospital, Taipei, 100225 Taiwan; 11 Division of Hematology and Internal Medicine, National Taiwan University Hospital, Taipei, 100225 Taiwan; 12 Graduate Institute of Clinical Medicine, National Taiwan University, Taipei, 106319 Taiwan; 13 Department of Family Medicine, Cardinal Tien Hospital, 362 Zhongzheng Rd., New Taipei, 231009 Taiwan; 14 Department of Family Medicine, E-Da Hospital, I-Shou University, 1 Yida Rd., Kaohsiung, 824005 Taiwan; 15 Bariatric and Metabolism International Surgery Center, E-Da Hospital, I-Shou University, Kaohsiung, 824005 Taiwan; 16 International Gaming Research Unit, Psychology Department, Nottingham Trent University, Nottingham, NG1 4FQ UK; 17 Biostatistics Consulting Center, National Cheng Kung University Hospital, College of Medicine, National Cheng Kung University, Tainan, 701401 Taiwan

**Keywords:** Adolescents, Young adults, Interpersonal sources, Media sources, Weight stigma sources

## Abstract

**Background:**

Weight stigma is pervasive, and it has a significant impact on the social, physical, and psychological health of an individual. Weight stigma is observed from several different sources. Therefore, the present study developed and validated a new instrument, the Weight Stigma Exposure Inventory (WeSEI), to assess different sources of observed weight stigma across interpersonal and non-interpersonal sources.

**Methods:**

The participants (n = 15,991) comprised Taiwanese young adults, Chinese adolescents, and Chinese young adults who completed paper-and-pencil and online surveys between September 2023 and December 2023. All participants provided demographic information, and completed the WeSEI, Weight Self-Stigma Questionnaire (WSSQ), and Perceived Weight Stigmatization Scale (PWSS). Exploratory factor analysis (EFA) and confirmatory factor analysis (CFA) were used to examine the factor structure of the WeSEI.

**Results:**

EFA and CFA results confirmed a seven-factor structure (television sources, traditional media sources, social media sources, parent sources, stranger sources, significant other sources, and friends sources) across 35 items of the WeSEI. Moreover, the WeSEI was supported by measurement invariance across subgroups (i.e., subsamples, gender, and weight status). Moreover, there were positive correlations between all seven factors of the WeSEI and the WSSQ and PWSS.

**Conclusion:**

The WeSEI appears to assess observed weight stigma from different sources, and had good reliability, validity, and invariance across various subsamples. The WeSEI may be useful in clinical practice and research for assessing exposure to weight stigma from different sources.

**Supplementary Information:**

The online version contains supplementary material available at 10.1186/s40337-024-01168-9.

## Abstract (highlights)

The present study adds to the empirical evidence regarding the contextual factors regarding weight stigma. The Weight Stigma Exposure Inventory (WeSEI) was developed to assess observed weight stigma from different sources, including interpersonal and non-interpersonal sources. Moreover, the WeSEI will be able to provide guidance to help reduce weight stigma across settings, as a useful tool for assessing its reduction. Moreover, future studies may use the WeSEI to better understand the relationships between weight stigma and other health outcomes or behaviors, such as eating disorders.

**Supplementary Information:**

The online version contains supplementary material available at 10.1186/s40337-024-01168-9.

## Introduction

Weight stigma is a consequence of discriminatory practices against individuals and negative beliefs towards other individuals based on their weight and appearance (e.g., that they are lazy, lack power, and are selfish) [1, 2]. Moreover, weight stigma has been identified as a social identity threat for individuals who are higher weight. Weight stigma increases the risk for many negative health consequences (e.g., emotional distress, cognitive deficits, eating disturbances) and is a barrier to health and well-being [1]. In addition to individuals who are higher weight, weight stigma has a negative impact on individuals across the weight spectrum [3]. Individuals’ misperceiving their actual weight status (i.e., higher weight, and underweight) is a potential risk factor among participants with average weight status that can increase psychological distress and the perception of weight stigma [3–5].

According to weight stigma evidence, its rapid spread is significant among individuals with higher weight [6–8]. Recent research found that almost 60% of participants (with higher weight) in Western countries (e.g., Australia, Canada, France, France, United States and United Kingdom) have experienced weight stigma [9]. Likewise, weight stigma is prevalent across Asia (especially, in China) [10]. The findings in China have shown that about 30% – 60% of the participants with higher weight have reported exposure to weight stigma and increased risk of experienced weight stigma [10, 11]. Moreover, a previous systematic review reported that there is increasing weight stigma research in Asia and that additional studies are needed to understand the extent of weight stigma [12].

Weight stigma includes negative stereotypical attitudes (e.g., beliefs that those who are higher weight are lazy, unattractive, etc.), and it might result in prejudice (e.g., negative attitudes towards those who are higher weight) and acts of discrimination (e.g., unfair treatment or social rejection) [6, 7]. Weight stigma is reported across several distinct settings and sources, including interpersonal sources (i.e., friends/peers, parents, significant others, and strangers) and non-interpersonal sources (i.e., television, movies, print media, and online media) [13]. Moreover, the effects of weight stigma might differ based on the sources of weight stigma [13]. Therefore, further research on contextual factors (i.e., interpersonal and non-interpersonal) is needed to understand weight stigma’s major sources and outcomes.

Scholars have operationally defined stigma as a social phenomenon that must include: (i) labelling, (ii) negative stereotyping, (iii) linguistic separation, and (iv) power asymmetry [14]. Stigma (with weight stigma being a common type) can be conceptualized into public stigma or personal stigma [7]. Public stigma is defined as the response of the public to those in specified groups (i.e., mental health illness) while personal stigma is defined as those in specified groups experiencing prejudice directly [7]. Moreover, personal stigma may also be described as having three essential features: (i) self-stigma (a reaction that accepts and endorses stereotypes within the self), (ii) perceived stigma (awareness of diminishing stereotypes, prejudice, and discrimination about the self), and (iii) experienced stigma (receiving prejudice and discrimination from other individuals), and these types of stigma may be observed through the three aforementioned components (i.e., stereotypes, prejudice, and discrimination) [7, 13, 15, 16].

However, distinguishing these different types of stigma may be impeded by their similar characteristics, particularly in distinguishing between perceived stigma and experienced stigma [13, 15, 17]. Stigmatizing attitudes among the public can influence both perceived stigma and experienced stigma; individuals may perceive concomitant negative beliefs, stigmatizing attitudes, and discriminatory behavior from the general population [18, 19]. Indeed, public stigma (i.e., others’ negative stereotypes) may be even more powerful than personal stigma [20]. Moreover, individuals (irrespective of whether they are higher weight or not) may observe weight stigma from the general public, and those who are higher weight may observe weight stigma and have experienced weight stigma.

Individuals who are higher weight experience weight stigma and discrimination in many different settings: in the workplace (e.g., from employers, co-workers), in healthcare settings (e.g., from doctors, nurses), in school and education environment (e.g., from friends, teachers), in personal relationships (i.e., parents, children), and in the media [2, 13]. Based on the Cyclic Obesity/Weight-Based Stigma (COBWEBS) model, weight stigma is characterized as social devaluation and denigration toward individuals who associate with higher weight through their experiences and practices [21]. Moreover, weight stigma can be defined as a potential stressor among individuals with higher weight [21, 22]. When individuals (who perceive themselves as being of higher weight) have stress about self-weight, stress could increase their negative behavior, emotionally and physiologically (e.g., increased cortisol secretion, increased food intake, and increased weight gain) [21]. Those who have experienced weight stigma face social problems (i.e., social isolation) and poor psychological health (e.g., reduced self-esteem, body image distress, disordered eating behaviors), which can contribute to poor physical health (e.g., lack of motivation to exercise, high blood pressure) [6, 16, 22]. Additionally, weight stigma can lead to short-term and long-term negative physical and psychological health outcomes [3, 13, 19, 22–26].

Weight stigma originates from many sources [27], the most common being family members, strangers, and the media. Various sources of weight stigma can contribute to different negative health effects [16, 28]. A previous study reported that being treated unfairly by family members was strongly associated with negative emotional affect (i.e., depression) [28]. However, weight-based discrimination by strangers had somewhat lower negative emotional affect [28]. Furthermore, media sources could increase weight-biased attitudes among individuals who perceive themseves as being higher weight such as body dissatisfaction and reduced self-esteem [29]. Therefore, research is needed to examine different sources of weight stigma, which might improve the development of effective interventions to decrease weight stigma.

Due to the high prevalence of weight stigma and its relation to adverse health outcomes [3, 19, 22, 24, 26, 30, 31], weight stigma researchers have developed numerous instruments related to weight stigma to understand the prevalence of weight stigma and to identify specific symptoms [31, 32]. In the extant literature, there are various instruments that assess weight stigma originating from external sources. These include the Stigmatizing Situations Inventory (SSI) [33] the Interpersonal Sources of Weight Stigma (ISWS) [8], the Physical Appearance Related Teasing Scale (PARTS) [34], the Treatment-based Experiences of Weight Stigma (STEWS) [35], and the Fat Microaggressions Scale [36]. Empirical evidence has also demonstrated that these instruments are widely recognized internationally as effective tools for evaluating external sources of stigmatization or discrimination against individuals who are higher weight [8, 33–36]. The SSI, ISWS and PARTS focus on interpersonal sources to assess individuals’ weight stigmatization and experiences of weight stigmatization [8, 33, 34]. The STEWS concerns individuals’ experiences of weight stigma in eating disorders from healthcare providers and peers [35]. The FMS assesses frequency of occurrence of experienced fat-microaggressions from both interpersonal and media sources among individuals with higher weight [36]. However, to the best of the authors’ knowledge, to date, there is no existing instrument assessing external sources of weight stigma from both interpersonal and non-interpersonal sources among individuals with various types of weight status (i.e., underweight, average weight, and higher weight). Moreover, there is no instrument that asks individuals to indicate how frequently they observe weight stigma in their daily lives and environments across interpersonal and non-interpersonal sources. Therefore, developing scales that could assess various ranges of contexts within which weight stigma occurs, and understanding how this stigma develops might contribute to reducing the impact of individuals’ weight-based stigmatizing experiences.

Previous findings have suggested the importance of contextual factors or sources of weight stigma in capturing the full extent of individuals’ experiences of weight stigmatization [16]. Sources of weight discrimination might predict the severity and health consequences of the stigma [16]. Pinpointing the specific source of weight stigma may facilitate its reduction by enabling the direct change of the specific source [30]. Therefore, a better assessment of sources of weight stigma might help bring about better understanding and prevention strategies.

Previous research has proposed that weight stigma could emanate from various sources, particularly media and interpersonal sources [8, 13, 28, 30, 37]. Therefore, it is important to develop an instrument to assess the frequency of exposure to weight stigma from various media and interpersonal sources. The Weight Stigma Exposure Inventory (WeSEI) was developed to address this literature gap by expanding the ISWS and FMS [8, 36]. More specifically, the ISWS asks participants to rate their frequency of exposure to weight stigma from interpersonal sources (i.e., family members, doctors, classmates, sales clerks at stores, friends, co-workers/colleagues, mother, spouse, servers at restaurants, nurses, general community, father, employers/supervisors, sister, dieticians/nutritionists, brother, teachers/processor, authority figures or police, mental health professionals, son, daughter, other) [8]. The FMS asks participants to rate their experiences of fat-microaggression from both interpersonal sources (i.e., healthcare providers and strangers) and non-interpersonal sources (i.e., social media, television shows, movies) among individuals with higher weight [36].

The WeSEI collapses these interpersonal sources to four important groups of people (i.e., parents and siblings, friends/peers, significant others, and strangers) and non-interpersonal sources of exposure (i.e., social media, traditional media, television series/movies) from the ISWS and FMS [8, 36]. Therefore, the purpose of the present study was to develop and validate the WeSEI to assess observed weight stigma across interpersonal and non-interpersonal sources.

## Methods

### Procedure and participants

The present study focused on young adults and adolescents because these cohorts are at high risk of experiencing weight stigma [10, 16, 27]. Moreover, although individuals who are higher weight are much more likely to be the target of wight stigma by others and experience psychological harm as a result of it, the present study did not just focus on individuals who are higher weight. This is because it is important to know whether individuals with different weight profiles interpret items on the WeSEI similarly.

The present study was approved by the following ethics committees: National Cheng Kung University Human Research Ethics Committee (Approval No.: NCKU HREC-E-111–563-2) and the Institute Review Board of Jiangxi Psychological Consultant Association (JXSXL-2023-SE0906) before beginning data collection. The data were collected from China (i.e., adolescents and university students) and Taiwan (i.e., university students), and the recruitment period was between September 2023 and December 2023. For Chinese adolescents, the participants were recruited from schools in China incorporating online surveys using a convenience sampling method. All participants and their parents or guardians provided written informed consent before participation, and the participants completed the online surveys in a computer classroom in their schools. The inclusion criteria for the Chinese adolescent sample included: (i) being aged 12–18 years, inclusive; (ii) having the ability to read and understand simplified Chinese characters; (iii) studying at a school in China; and (iv) providing voluntary agreement for study participation from both participants and their parents/guardians. For Chinese and Taiwanese university students, the participants were recruited via a link to a self-report online survey (i.e., using *SoJump* for the sample in China and *SurveyMonkey* for the sample in Taiwan) using a convenience sampling method. The inclusion criteria for Chinese and Taiwanese university students included: (i) being age ≥ 18 years for Chinese participants and being aged ≥ 20 years for Taiwanese participants; (ii) studying at a university in China or Taiwan; (iii) being able to understand and read simplified or traditional Chinese characters, and (iv) providing voluntary agreement for study participation. All participants provided demographic information and completed the WeSEI, Weight Self-Stigma Questionnaire (WSSQ), and Perceived Weight Stigmatization Scale (PWSS). It took approximately 15 min to complete all the survey questions. It should also be noted that the online surveys could only be submitted if all the items had been answered. Therefore, there were no missing data in the present study.

### Measures

#### Demographic information

Demographic information was collected including questions relating to age, sex, self-reported weight (in kilograms) and height (in centimeters). Moreover, weight status was based on body mass index (BMI) calculated using weight divided by squared height (in meters).

#### Weight Stigma Exposure Inventory (WeSEI)

The WeSEI was constructed by the research team and consulted experts in psychology and weight stigma. (Details of how the WeSEI items were generated and the first draft of the WeSEI items are reported in Supplementary Material A and Supplementary Table S1). The WeSEI asks participants to rate their frequency of exposure to weight-based stigma by media and interpersonal sources. The scale comprises 35 items categorized into seven domains (each with five items): television sources (Factor 1), traditional media sources (Factor 2), parent sources (Factor 3), stranger sources (Factor 4), social media sources (Factor 5), significant others sources (Factor 6), and friend sources (Factor 7) (these 35 items are listed in Supplementary Table S2). WeSEI items are rated on a five-point Likert scale (1 = Never; 2 = Seldom; 3 = Sometimes; 4 = Often; 5 = Almost always). A total score of the WeSEI is calculated by averaging the items in each factor. A higher score on each factor indicates higher rates of observed weight stigma in that source.

#### Weight Self-Stigma Questionnaire (WSSQ)

The WSSQ asks participants to rate their perception of weight-based self-stigma [38]. The scale comprises 12 items categorized into two domains (i.e., self-devaluation and fear of enacted stigma). WSSQ items are rated on a five-point Likert scale (1 = Strongly disagree; 2 = Disagree; 3 = Neutral; 4 = Agree; 5 = Strongly agree). A sample item of the self-devaluation domain is *“I caused my weight problems”*; a sample item of the fear of enacted stigma domain is *“People discriminate against me because I’ve had weight problems”.* The total WSSQ score is calculated by summing the total of items; a higher score indicates higher rates of weight-related self-stigma. The WSSQ has been translated into Chinese (α = 0.88 for the total score; α = 0.78 for the self-devaluation domain; α = 0.88 for the fear of enacted stigma domain) [39]. The internal consistency of WSSQ in the present study was very good to excellent among both the Chinese sample (α = 0.95 [young adults] and 0.97 [adolescents] for total score; α = 0.90 [young adults] and 0.93 [adolescents] for self-devaluation domain; α = 0.94 [young adults] and 0.97 [adolescents] for the fear of enacted stigma domain) and the Taiwanese sample (α = 0.93 for total score; α = 0.87 for self-devaluation domain; α = 0.89 for the fear of enacted stigma domain).

#### Perceived Seight Stigma Scale (PWSS)

The PWSS asks participants to rate their perceived weight stigma [40]. The scale comprises 10 items which are rated dichotomously (0 = No; 1 = Yes) [41]. A sample item of the PWSS is *“You are treated with less respect than others”.* The total score of the PWSS is calculated by summing the 10-item score; a higher score indicates higher perceived weight stigma [42]. Moreover, the PWSS has been translated into Chinese (α = 0.84) [42], The internal consistency of PWSS in the present study was very good in both the Chinese sample (α = 0.86 [young adults] and 0.91 [adolescents]) and the Taiwanese sample (α = 0.87).

### Statistical analysis

First, descriptive statistics were conducted to examine participants’ characteristics and average scores of WSSQ (including self-devaluation and fear of enacted stigma domains) and PWSS. Subsequently, the factor structure of the WeSEI was examined in the following two steps: exploratory factor analysis (EFA) and confirmatory factor analysis (CFA). In the first step, analysis was conducted to see if the data supported the proposed factor structure of the WeSEI using EFA. Before performing EFA, sampling adequacy was determined using the Kaiser–Meyer–Olkin test (KMO-test) and Bartlett’s test of sphericity. A value of KMO-test > 0.08 and Bartlett’s test significant level of *p* < 0.05 indicate acceptable sampling for performing EFA [43, 44].

Moreover, the Mardia test was applied for multivariate normality analysis within the data. As shown in Supplementary Table S3, the Mardia test presented multivariate skewness and kurtosis violating the multinormal distribution (*p* < 0.001). Therefore, principal axis factor with polychronic correlation was performed for the EFA [45] to extract factors in the WeSEI, using the oblique rotation method (i.e., *oblimin*). The scree plot was first applied with eigenvalues to determine the number of factors to extract in the WeSEI. Eigenvalues > 1 indicate adequate factors to extract [46]. The total variance (> 60%) indicated the adequate number of factors to retain [45]. Factor pattern matrix analysis was additionally performed to determine the number of items in each factor after oblique rotation. The values of factor loadings of > 0.4 were used as an indication of an adequate number of items in each factor [47].

In the second step, CFA was performed to confirm whether the factor structure obtained from the EFA found in the first step was the same. As aforementioned, multivariate skewness and kurtosis violated the multivariate normal distribution (*p* < 0.001) (Supplementary Table S3). Therefore, diagonally weighted least squares (DWLS) estimator was applied to examine factor structure in the CFA [48, 49]. The model fit was evaluated using χ^2^ tests with fit indices including comparative fit index (CFI), Tucker-Lewis index (TLI), root mean error of approximation (RMSEA), and standardized root mean square residual (SRMR). Non-significant χ^2^, values of both CFI and TLI > 0.95 and values of both RMSEA and SRMR < 0.08 are regarded as satisfactory factor structure [49, 50]. Moreover, factor loadings derived from CFA, were performed to examine all items of the WeSEI. A value of standardized factor loadings greater than 0.4 is considered acceptable [51].

After the factor structure of the WeSEI was confirmed by CFA, the confirmed factor structure was further examined using multi-group CFA (MGCFA) across different subgroups: sample (Taiwanese young adults vs. Chinese adolescents vs. Chinese young adults), gender (male vs. female), and weight status (higher weight vs. non-higher weight) subgroups. The present study proposed three series of nested MGCFAs for measurement invariance analysis: (i) configural invariance (where similar factor structure is measured across subgroups); (ii) metric invariance (where similar factor loadings are measured across subgroups); and (iii) scalar invariance (where similar item thresholds are measured across subgroups). The differences in χ^2^ test and fit indices (i.e., ∆CFI, ∆RMSEA, ∆SRMR) between two nested models were used to evaluate measurement invariance of the WeSEI across subgroups. Additionally, non-significant χ^2^ and values of fit indices (i.e., ∆CFI < 0.01, ∆RMSEA < 0.015, ∆SRMR < 0.03 [for factor loadings], and ∆SRMR < 0.01 [for intercepts]) indicate measurement invariance across subgroups [52].

In addition, average variance extracted (AVE) was performed to examine the convergent validity of the same WeSEI factor. A value of AVE > 0.5 is regarded as an acceptable level [53]. Henseler’s heterotrait-monotrait ratio of correlations (HTMT) was further performed to examine the discriminant validity between factors of the WeSEI, with a value of HTMT < 0.85 regarded as acceptable [54].

Pearson correlations were performed to examine concurrent validity between each factor of the WeSEI with the WSSQ and PWSS among Taiwanese young adults, Chinese adolescents, and Chinese young adults. The magnitudes of Pearson correlation coefficients are defined as follows: 0.10–0.29 are considered low; 0.30–0.49 are considered moderate; and ≥ 0.5 are considered high [55]. Finally, the internal consistency of the WeSEI was assessed using both Cronbach’s alpha (α) and McDonald’s omega (ω), and both α and ω coefficient scores higher than 0.7 are regarded as satisfactory [56, 57]. Moreover, independent *t*-tests were conducted to examine if the non-higher weight group had different WeSEI scores from the higher weight group. The present study performed statistical analyses (e.g., independent *t*-tests) using IBM SPSS 23.0 [58] to provide descriptive statistics and examine concurrent validity. EFA and internal consistency were conducted using JASP 0.17.2.1 [59]; CFAs including MGCFA were performed using the *lavaan* package in R software [60]; AVE and HTMT were calculated using *semTools* package in R software [61].

## Results

The entire sample with three subsamples comprised 15,991 participants (887 Taiwanese young adults, 11,123 Chinese adolescents, and 3,981 Chinese young adults). The response rates on online surveys were 88.7% among Taiwanese young adults, 94.4% among Chinese adolescents, and 99.5% among Chinese young adults. Additionally, the present study used attention checks questions in the online surveys to ensure data quality. Table 1 shows the participants’ characteristics. In brief, the total study participants (46.4% females) had a mean age of 17.74 years (SD = 3.42). A total of 55.4% of participants were higher weight (based on their BMI). Moreover, the total sample had a mean score on the WSSQ of 2.25 (SD ± 0.94) for Factor 1 (self-devaluation subscale), and 2.13 (SD ± 0.97) for Factor 2 (fear of enacted stigma subscale); and a mean score on the PWSS of 0.14 (SD ± 0.25).

**Table 1:** Participants’ characteristics (N = 15,991)

	Entire sample (N = 15,991)	Taiwanese young adults (n = 887)	Chinese adolescents (n = 11,123)	Chinese young adults (n = 3,981)
Age (in years); Mean (SD)/ range	17.74 (3.42)/ 12–40	28.82 (6.06)/ 20–40	16.36 (0.78)/ 12–18	19.20 (1.38)/ 18–40
BMI (kg/m^2^); Mean (SD)/ range	28.60 (10.96)/ 10–50	22.82 (4.00)/ 14–49	29.93 (11.54)/ 10–50	26.15 (9.42)/ 10–50
Sex (female); n (%)	7424 (46.4)^a^	565 (63.7)^a^	4573 (41.1)	2286 (57.4)
Weight status; n (%)				
Higher weight	8852 (55.36)	364 (41.04)	6708 (60.31)	1780 (44.71)
Average weight	5818 (36.38)	469 (52.87)	3640 (32.72)	1709 (42.93)
Underweight	1321 (8.26)	54 (6.09)	775 (6.97)	492 (12.36)
WSSQF1 score; Mean (SD)	2.25 (0.94)	2.51 (0.88)	2.24 (0.96)	2.22 (0.91)
WSSQF2 score; Mean (SD)	2.13 (0.97)	2.29 (0.87)	2.13 (0.99)	2.10 (0.92)
PWSS score; Mean (SD)	0.14 (0.25)	0.13 (0.23)	0.14 (0.26)	0.13 (0.23)

*Notes*: BMI = body mass index; WSSQF1 = Weight Self-Stigma Questionnaire Self-devaluation subscale; WSSQF2 = Weight Self-Stigma Questionnaire Fear of enacted stigma subscale; PWSS = Perceived Weight Stigma Scale. WSSQF1 and WSSQF2 scores are presented using standardized scale between 1 and 5; PWSS score using standardized scale between 0 and 1

^a^Three participants reported their sex as ‘Other’

**Fig. 1:**
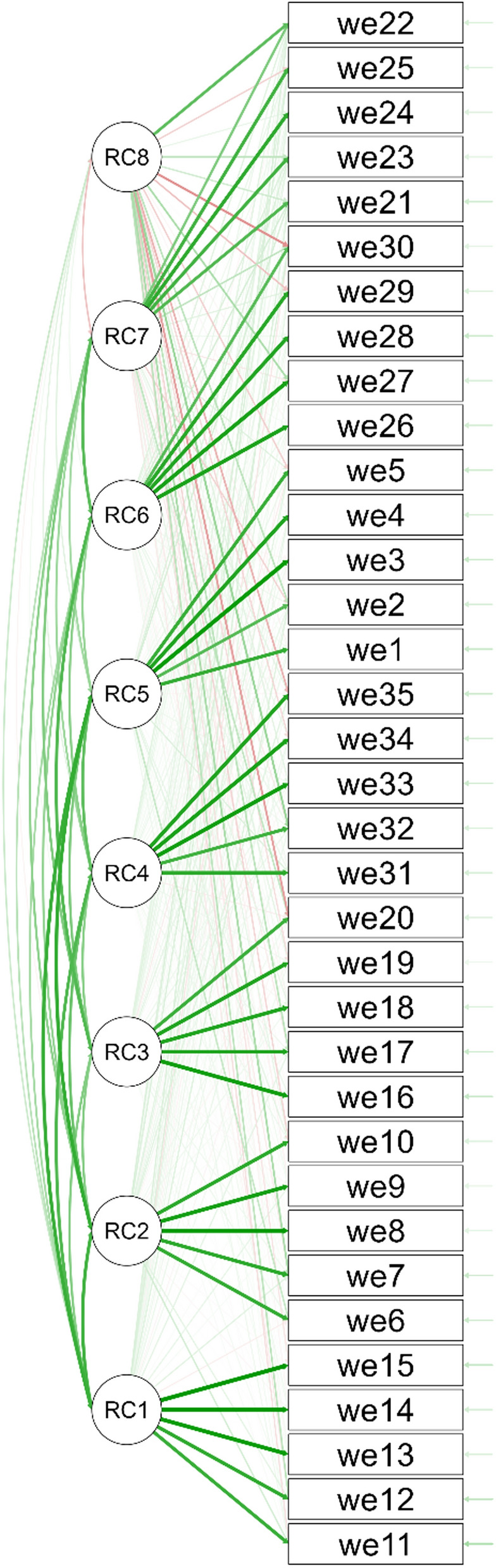
Exploratory factor analysis results of the Weight Stigma Exposure Inventory (WeSEI) using the Taiwanese young adult sample (n = 887). Notes. Green lines indicate positive relationship; red lines negative relationship. Thicker lines indicate stronger correlations. RC1 = TV source; RC2 = Traditional media source; RC3 = Parent source; RC4 = Stranger source; RC5 = Social media source; RC6 = Significant others source; RC7 = Friend source; RC8 was not used as it associated with all items and factors weakly

**Fig. 2:**
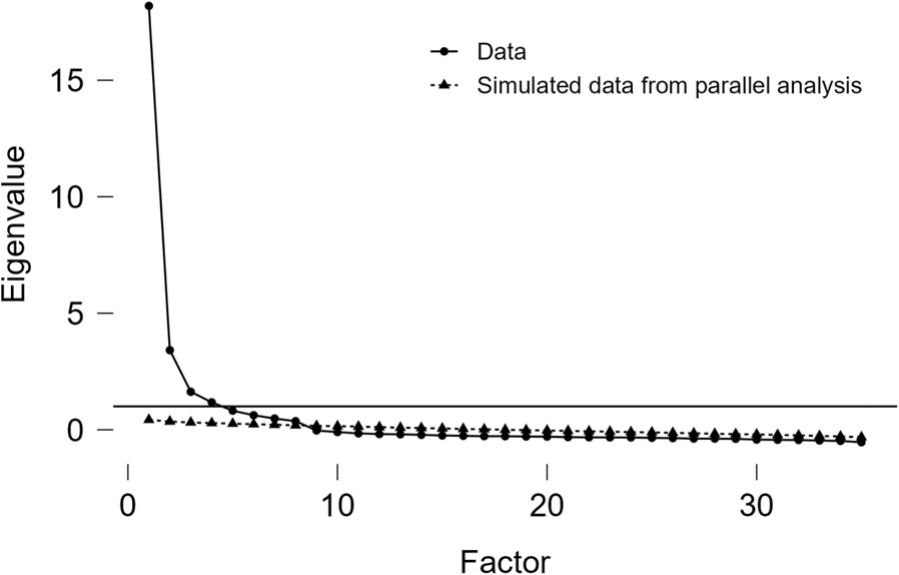
Scree plot with parallel analysis for the Weight Stigma Exposure Inventory (WeSEI) using Taiwanese young adult sample (n = 887)

Factor structure of the WeSEI was first explored using EFA with the sample of Taiwanese young adults, and an eight-factor structure was found. More specifically, the value of KMO was 0.964 and Bartlett’s test was significant, indicating acceptable data for performing EFA. According to the scree plot with parallel analysis (Fig. 1), eight factors were extracted that explained a total of 83.6% of the variance (Factor 1 = 12.9%; Factor 2 = 12.4%; Factor 3 = 11.5%; Factor 4 = 11.4%; Factor 5 = 11.3%; Factor 6 = 11.0%; Factor 7 = 8.8%; and Factor 8 = 4.4%). However, the eighth factor was deleted because all factor loadings for this factor were relatively weak (only one item had a loading over 0.5, and the remaining items’ loadings were smaller than 0.4; Fig. 2). Therefore, seven factors (factor loadings presented in Table 2) were used for the WeSEI structure for further analyses. Moreover, the seven factors were named as television sources (Factor 1), traditional media sources (Factor 2), parent sources (Factor 3), stranger sources (Factor 4), social media sources (Factor 5), significant others sources (Factor 6), and friend sources (Factor 7).

**Table 2:** Factor loading findings from exploratory factor analysis for the Weight Stigma Exposure Inventory (WeSEI) using Taiwanese young adult sample (n = 887)

Item	Factor 1	Factor 2	Factor 3	Factor 4	Factor 5	Factor 6	Factor 7	Factor 8
we1	0.002	0.075	0.044	– 0.036	0.729	0.055	0.037	– 0.051
we2	0.121	0.060	0.094	0.043	0.605	0.062	– 0.162	0.242
we3	0.026	– 0.029	0.062	0.047	0.887	– 0.032	0.006	0.010
we4	0.069	0.082	– 0.022	0.025	0.799	0.010	0.011	– 0.029
we5	0.048	0.103	– 0.067	0.072	0.711	0.003	0.117	– 0.176
we6	– 0.061	0.733	0.064	– 0.008	0.210	0.027	0.026	– 0.004
we7	0.107	0.756	0.053	0.030	– 0.021	0.028	– 0.074	0.303
we8	0.030	0.900	0.026	0.048	– 0.008	– 0.018	0.008	0.011
we9	0.085	0.844	– – 0.025	0.011	0.028	0.056	0.023	– 0.049
we10	0.020	0.762	0.007	0.063	0.102	– 0.032	0.092	– 0.157
we11	0.709	0.164	0.026	– 0.012	0.061	0.046	0.022	– 0.124
we12	0.749	0.101	0.042	0.000	– 0.001	0.009	– 0.079	0.242
we13	0.879	0.006	0.014	0.040	0.014	– 0.034	0.044	– 0.009
we14	0.920	– 0.025	0.012	0.027	0.055	0.015	0.009	– 0.037
we15	0.920	– 0.014	0.002	0.014	0.017	0.016	0.025	– 0.104
we16	0.005	0.019	0.859	0.014	0.081	0.022	– 0.018	0.002
we17	0.098	0.032	0.807	0.059	0.007	0.002	– 0.075	0.244
we18	0.010	0.057	0.804	0.033	0.016	0.092	– 0.040	0.055
we19	0.055	– 0.032	0.800	0.059	0.001	0.029	0.101	– 0.129
we20	– 0.038	0.054	0.701	– 0.066	0.007	0.021	0.225	– 0.344
we21	0.126	0.037	0.047	0.022	0.145	0.153	0.620	0.177
we22	0.132	0.041	0.061	0.111	0.077	0.116	0.459	0.577
we23	0.006	0.002	0.101	0.067	0.124	0.125	0.673	0.222
we24	0.075	0.082	0.050	0.074	0.041	0.055	0.748	0.084
we25	0.012	0.133	0.102	0.058	– 0.034	0.078	0.680	– 0.169
we26	– 0.039	0.012	0.056	0.053	0.068	0.819	0.033	– 0.032
we27	0.053	0.019	0.010	0.020	0.046	0.829	– 0.087	0.299
we28	0.045	– 0.006	0.072	0.047	– 0.021	0.791	0.058	0.010
we29	0.092	0.021	0.044	0.011	– 0.000	0.768	0.107	– 0.221
we30	– 0.090	0.132	0.088	0.032	– 0.011	0.595	0.230	– 0.398
we31	0.006	0.021	0.041	0.761	0.126	0.076	– 0.018	0.059
we32	0.076	0.015	0.073	0.689	0.054	0.075	– 0.080	0.360
we33	0.017	– 0.030	0.060	0.858	0.058	0.024	– 0.014	0.108
we34	0.063	0.068	– 0.014	0.828	0.009	– 0.005	0.070	– 0.147
we35	0.045	0.111	– 0.008	0.799	– 0.036	– 0.017	0.098	– 0.279

*Notes*: Eigenvalue from real data/mean of simulations = 18.19/0.43 (F1), 3.42/0.35 (F2), 1.63/0.31 (F3), 1.18/0.29 (F4), 0.82/0.26 (F5), 0.62/0.24 (F6), 0.48/0.21 (F7), and 0.37/0.19 (F8). Variance explained for each factor after rotation was: 12.9% (F1), 12.4% (F2), 11.5% (F3), 11.4% (F4), 11.3% (F5), 11.0% (F6), 8.8% (F7), and 4.4% (F8); cumulative explained variance = 83.6%. Kaiser–Meyer–Olkin test = 0.946; χ^2^ (df) of Bartlett’s test = 41,533.50 (595); *p*-value of Bartlett’s test < 0.001. Extraction method was principal axis functioning with polychronic correlation matrix; oblique rotation method was oblimin. Number of factors was decided using parallel analysis. Factor 1 = TV source; Factor 2 = Traditional media source; Factor 3 = Parent source; Factor 4 = Stranger source; Factor 5 = Social media source; Factor 6 = Significant others source; Factor 7 = Friend source; Factor 8 was not used

The seven-factor structure of the WeSEI was confirmed using CFA with two different subsamples (i.e., Chinese adolescents and young adults). Because multivariate skewness and kurtosis violated the multinormal distribution (*p* < 0.001) (Supplementary Table S3), the DWLS was performed for the CFA. Table 3 demonstrates the CFA results for the WeSEI. For Chinese adolescents, CFA findings showed an acceptable fit (χ^2^ [df] = 1176.355 [539]; CFI = 1.000; TLI = 1.000; RMSEA = 0.010; SRMR = 0.018), except for the significant χ^2^ (< 0.001). Moreover, all WeSEI items had acceptable factor loadings (Factor 1 = 0.903–0.956; Factor 2 = 0.930–0.950; Factor 3 = 0.905–0.938; Factor 4 = 0.932–0.958; Factor 5 = 0.872–0.928; Factor 6 = 0.934–0.961; and Factor 7 = 0.919–0.950). For Chinese young adults, CFA findings also showed an acceptable fit (χ^2^ [df] = 2241.160 [539]; CFI = 0.997; TLI = 0.997; RMSEA = 0.028; SRMR = 0.039), except for the significant χ^2^ (< 0.001). Similarly, all WeSEI items had acceptable factor loadings (Factor 1 = 0.872–0.958; Factor 2 = 0.921–0.944; Factor 3 = 0.871–0.916; Factor 4 = 0.883–0.947; Factor 5 = 0.832–0.902; Factor 6 = 0.892–0.951; and Factor 7 = 0.862–0.931) (Table 4).

**Table 3:** Confirmatory factor analysis results for the Weight Stigma Exposure Inventory (WeSEI) using mainland Chinese adolescents (n = 11,123) and young adult samples (n = 3,981)

	Chinese adolescents	Chinese young adults	Suggested cutoff
*Fit indices*			
χ^2^ (*df*)	1176.355 (539)	2241.160 (539)	–
*p*-value of χ^2^	< 0.001	< 0.001	Nonsignificant
CFI	1.000	0.997	> 0.95
TLI	1.000	0.997	> 0.95
RMSEA (90% CI)	0.010 (0.010, 0.011)	0.028 (0.027, 0.029)	< 0.08
SRMR	0.018	0.039	< 0.08
*Factor loadings*			
we1 (Factor 5)	0.872	0.835	> 0.4
we2 (Factor 5)	0.879	0.832	> 0.4
we3 (Factor 5)	0.890	0.863	> 0.4
we4 (Factor 5)	0.927	0.900	> 0.4
we5 (Factor 5)	0.928	0.902	> 0.4
we6 (Factor 2)	0.930	0.932	> 0.4
we7 (Factor 2)	0.933	0.921	> 0.4
we8 (Factor 2)	0.941	0.937	> 0.4
we9 (Factor 2)	0.950	0.944	> 0.4
we10 (Factor 2)	0.941	0.935	> 0.4
we11 (Factor 1)	0.956	0.958	> 0.4
we12 (Factor 1)	0.903	0.872	> 0.4
we13 (Factor 1)	0.954	0.943	> 0.4
we14 (Factor 1)	0.928	0.925	> 0.4
we15 (Factor 1)	0.919	0.913	> 0.4
we16 (Factor 3)	0.936	0.916	> 0.4
we17 (Factor 3)	0.932	0.902	> 0.4
we18 (Factor 3)	0.938	0.915	> 0.4
we19 (Factor 3)	0.935	0.909	> 0.4
we20 (Factor 3)	0.905	0.871	> 0.4
we21 (Factor 7)	0.950	0.931	> 0.4
we22 (Factor 7)	0.926	0.862	> 0.4
we23 (Factor 7)	0.945	0.918	> 0.4
we24 (Factor 7)	0.942	0.902	> 0.4
we25 (Factor 7)	0.919	0.866	> 0.4
we26 (Factor 6)	0.958	0.951	> 0.4
we27 (Factor 6)	0.955	0.919	> 0.4
we28 (Factor 6)	0.961	0.943	> 0.4
we29 (Factor 6)	0.958	0.925	> 0.4
we30 (Factor 6)	0.934	0.892	> 0.4
we31 (Factor 4)	0.953	0.947	> 0.4
we32 (Factor 4)	0.932	0.883	> 0.4
we33 (Factor 4)	0.950	0.936	> 0.4
we34 (Factor 4)	0.958	0.940	> 0.4
we35 (Factor 4)	0.947	0.926	> 0.4

*Notes.* The tested factor structure is a seven-factor structure: Factor 1 = TV source; Factor 2 = Traditional media source; Factor 3 = Parent source; Factor 4 = Stranger source; Factor 5 = Social media source; Factor 6 = Significant others source; Factor 7 = Friend source. *df* = degrees of freedom; CFI = comparative fit index; TLI = Tucker-Lewis index; RMSEA = root mean square error of approximation; CI = confidence interval; SRMR = standardized root mean square residual

Diagonally weighted least squares estimation was used for the confirmatory factor analysis

**Table 4:** Measurement invariance of the Weight Stigma Exposure Inventory (WeSEI) across different subgroups (sample subgroups; sex subgroups; weight status subgroups)

Subgroup	Nested models			Model comparisons	
Fit indices	M0 Configural	M1 Metric	M2 Scalar	M0 vs. M1	M1 vs. M2
*Sample (Taiwanese young adults [n = 887], Chinese adolescents [n = 11,123], and Chinese young adults [n = 3,981])*					
χ^2^ (*df*) [nested models] or Δχ^2^ (Δ*df*) [model comparisons]	4825.024 (1617)	6231.681 (1673)	7364.480 (1729)	1406.7 (56)	1132.8 (56)
*p*-value	< 0.001	< 0.001	< 0.001	< 0.001	< 0.001
CFI [nested models] or ΔCFI [model comparisons]	0.999	0.998	0.998	−0.001	0.000
TLI [nested models] or ΔTLI [model comparisons]	0.999	0.998	0.998	−0.001	0.000
RMSEA [nested models] or ΔRMSEA [model comparisons]	0.019	0.023	0.025	0.004	0.002
SRMR [nested models] or ΔSRMR [model comparisons]	0.025	0.027	0.028	0.002	0.001
Sex (males [n = 8,564] and females [n = 7,424])					
χ^2^ (df) [nested models] or Δχ^2^ (Δdf) [model comparisons]	4290.430 (1078)	5742.370 (1106)	6091.747 (1134)	1451.9 (28)	349.4 (28)
*p*-value	< 0.001	< 0.001	< 0.001	< 0.001	< 0.001
CFI [nested models] or ΔCFI [model comparisons]	0.999	0.998	0.998	−0.001	0.000
TLI [nested models] or ΔTLI [model comparisons]	0.999	0.998	0.998	−0.001	0.000
RMSEA [nested models] or ΔRMSEA [model comparisons]	0.019	0.023	0.023	0.004	0.000
SRMR [nested models] or ΔSRMR [model comparisons]	0.025	0.029	0.030	0.004	0.001
*Weight status (higher weight [n = 8,840] and non-higher weight [n = 7,151])*					
χ^2^ (df) [nested models] or Δχ^2^ (Δdf) [model comparisons]	4231.093 (1078)	4420.071 (1106)	4461.231 (1134)	189.0 (28)	41.2 (28)
*p*-value	< 0.001	< 0.001	< 0.001	< 0.001	0.052
CFI [nested models] or ΔCFI [model comparisons]	0.999	0.999	0.999	0.000	0.000
TLI [nested models] or ΔTLI [model comparisons]	0.999	0.999	0.999	0.000	0.000
RMSEA [nested models] or ΔRMSEA [model comparisons]	0.019	0.019	0.019	0.000	0.000
SRMR [nested models] or ΔSRMR [model comparisons]	0.027	0.027	0.027	0.000	0.000

*df* = degrees of freedom; CFI = comparative fit index; TLI = Tucker-Lewis index; RMSEA = root mean square error of approximation; SRMR = standardized root mean square residual

After confirming the seven-factor structure for the WeSEI, measurement invariance across subsamples (i.e., Taiwanese young adults, Chinese adolescents, and Chinese young adults) were tested. The findings showed that the seven-factor structure of the WeSEI was supported given the acceptable fit indices shown in the measurement invariance findings. More specifically, except for significant χ^2^ tests and χ^2^ difference tests, (i) all configural models had satisfactory fit indices (all CFI = 0.999; all TLI = 0.999; all RMSEA = 0.019; SRMR = 0.025 to 0.027); (ii) metric invariance models had similar fit indices to the configural models (ΔCFI and ΔTLI = −0.001, ΔRMSEA = 0.004, and ΔSRMR = 0.002 for subsample group comparisons; ΔCFI and ΔTLI = −0.001, ΔRMSEA = 0.004, and ΔSRMR = 0.004 for sex group comparisons; and ΔCFI, ΔTLI, ΔRMSEA, and ΔSRMR = 0.000 for weight status group comparisons); and (iii) scalar invariance models had similar fit indices to the metric invariance models (ΔCFI and ΔTLI = 0.00, ΔRMSEA = 0.002, and ΔSRMR = 0.001 for subsample group comparisons; ΔCFI, ΔTLI, and ΔRMSEA = 0.000, and ΔSRMR = 0.001 for sex group comparisons; and ΔCFI, ΔTLI, ΔRMSEA, and ΔSRMR = 0.000 for weight status group comparisons).

For discriminant validity (Table 5), HTMT analysis showed that some factors had values larger than 0.85 or 0.90, indicating that some factors might not be discriminant from each other. More specifically, in the entire sample, Factor 2 (traditional media sources) might not be discriminant from Factor 5 (social media sources) (HTMT = 0.871); Factor 3 (parent sources) might not be discriminant from Factor 6 (significant others sources) (HTMT = 0.874) or Factor 7 (friend sources) (HTMT = 0.892); Factor 6 (significant others sources) might not be discriminant from Factor 7 (friend source) (HTMT = 0.918). Among the Chinese adolescent subsample, Factor 1 (television sources) might not be discriminant from Factor 2 (traditional media sources) (HTMT = 0.860); Factor 2 (traditional media sources) might not be discriminant from Factor 5 (social media sources) (HTMT = 0.886); Factor 3 (parent sources) might not be discriminant from Factor 6 (significant other sources) (HTMT = 0.895); Factor 3 (parent sources) might not be discriminant from Factor 7 (friend sources) (HTMT = 0.908); Factor 6 (significant other sources) might not be discriminant from Factor 7 (friend sources) (HTMT = 0.923). Among the Chinese young adult subsample, Factor 3 (parent sources) might not be discriminant from Factor 7 (friend sources) (HTMT = 0.882); and Factor 6 (significant other sources) might not be discriminant from Factor 7 (friend sources) (HTMT = 0.925). However, the discriminant validity was fully supported for the WeSEI factors among the Taiwanese young adult subsample.

**Table 5:** Convergent and discriminant validity of the Weight Stigma Exposure Inventory (WeSEI) using average variance extracted (AVE) and Henseler’s heterotrait-monotrait ratio of correlations (HTMT) (N = 15,991)

	F1	F2	F3	F4	F5	F6	F7
*Entire sample (N* = *15,991)*							
F1	(0.860)						
F2	0.821	(0.872)					
F3	0.716	0.762	(0.839)				
F4	0.797	0.749	0.755	(0.878)			
F5	0.834	0.871	0.701	0.763	(0.789)		
F6	0.671	0.744	0.874	0.770	0.672	(0.878)	
F7	0.728	0.773	0.892	0.810	0.724	**0.918**	(0.840)
*Taiwanese young adults (n* = *887)*							
F1	(0.755)						
F2	0.741	(0.746)					
F3	0.494	0.525	(0.681)				
F4	0.697	0.641	0.509	(0.718)			
F5	0.763	0.796	0.536	0.674	(0.673)		
F6	0.401	0.487	0.686	0.514	0.471	(0.655)	
F7	0.544	0.624	0.666	0.615	0.604	0.777	(0.629)
*Chinese adolescents (n* = *11,123)*							
F1	(0.867)						
F2	0.860	(0.882)					
F3	0.762	0.782	(0.864)				
F4	0.804	0.775	0.790	(0.898)			
F5	0.849	0.886	0.721	0.771	(0.809)		
F6	0.729	0.763	0.895	0.816	0.695	(0.909)	
F7	0.772	0.789	**0.908**	0.845	0.737	**0.923**	(0.876)
*Chinese young adults (n* = *3,981)*							
F1	(0.848)						
F2	0.736	(0.871)					
F3	0.639	0.739	(0.816)				
F4	0.767	0.687	0.703	(0.856)			
F5	0.792	0.838	0.665	0.734	(0.750)		
F6	0.576	0.734	0.846	0.698	0.644	(0.858)	
F7	0.636	0.739	0.882	0.742	0.689	**0.925**	(0.801)

*Notes.* AVEs are presented in diagonal line and in parentheses; HTMTs in lower triangular matrix. All AVEs showed that the seven factors have good convergent validity (i.e., > 0.5) across all subsamples and the entire sample. Factors with probable discriminant validity problem are in shadows (using HTMT 0.85 as cutoff) or in bold (using HTMT 0.90 as cutoff)

Factor 1 = TV source; Factor 2 = Traditional media source; Factor 3 = Parent source; Factor 4 = Stranger source; Factor 5 = Social media source; Factor 6 = Significant others source; Factor 7 = Friend source

Supplementary Table S4 shows the inter-factor correlations of the WeSEI, using the Taiwanese young adult subsample. The results showed that there was a high correlation between Factor 1 and Factor 2 (*r* = 0.658), Factor 1 and Factor 5 (*r* = 0.680), and Factor 2 and Factor 5 (*r* = 0.698). Moreover, the WeSEI items showed very good to excellent internal consistency (Cronbach’s α = 0.891–0.939, McDonald’s ω = 0.894–0.940) among Taiwanese young adult subsample. Supplementary Table S5 shows the inter-factor correlations of the WeSEI, using the Chinese adolescent subsample. The results showed that there was a high correlation between Factor 3 and Factor 7 (*r* = 0.907), and between Factor 6 and Factor 7 (*r* = 0.922). Moreover, the WeSEI items showed excellent internal consistency (both Cronbach’s α and McDonald’s ω = 0.955–0.980) among the Chinese adolescent subsample. Supplementary Table S6 shows the inter-factor correlations of the WeSEI, using a Chinese young adult subsample. The results showed that there was a high correlation between Factor 6 and Factor 7 (*r* = 0.922). Moreover, the WeSEI items showed excellent internal consistency (both Cronbach’s α and McDonald’s ω = 0.938–0.971) among the Chinese young adult subsample.

After ensuring the internal validity of the WeSEI, Table 6 shows the concurrent validity of the WeSEI using correlations with WSSQ and PWSS. All the WeSEI factors were statistically significant (*p* < 0.001) and had small to moderate level of correlations with WSSQ (both Factor 1, self-devaluation, and Factor 2, fear of enacted stigma), and PWSS scores among the entire sample, Taiwanese young adults, Chinese adolescents, and Chinese young adults. Among the entire sample, the WeSEI had moderate associations with WSSQ Factor 1 (self-devaluation; range between 0.519 and 0.555) and WSSQ Factor 2 (fear of enacted stigma; range between 0.517–0.585 for Factor 2) scores. However, the WeSEI had relatively weak associations with PWSS (range between 0.238 and 0.281). Among the Taiwanese young adult subsample, the WeSEI had relatively weak associations with WSSQ Factor 1 (self-devaluation; range between 0.244 and 0.360), WSSQ Factor 2 (fear of enacted stigma; range between 0.225 and 0.407), and PWSS (range between 0.186 and 0.317) scores.

**Table 6:** Concurrent validity of the Weight Stigma Exposure Inventory (WeSEI) with Weight Self-Stigma Questionnaire and Perceived Weight Stigma Scale (N = 15,991)

	F1	F2	F3	F4	F5	F6	F7
*Entire sample (N* = *15,991)*							
WSSQF1	0.519	0.534	0.553	0.539	0.534	0.539	0.555
WSSQF2	0.517	0.550	0.584	0.552	0.529	0.579	0.585
PWSS	0.238	0.258	0.278	0.259	0.247	0.266	0.281
*Taiwanese young adults (n* = *887)*							
WSSQF1	0.246	0.271	0.360	0.237	0.305	0.281	0.244
WSSQF2	0.225	0.272	0.407	0.248	0.295	0.351	0.314
PWSS	0.186	0.240	0.317	0.249	0.258	0.269	0.274
*Chinese adolescents (n* = *11,123*)							
WSSQF1	0.558	0.559	0.572	0.569	0.558	0.560	0.578
WSSQF2	0.560	0.570	0.598	0.586	0.556	0.595	0.603
PWSS	0.250	0.256	0.271	0.260	0.255	0.263	0.279
*Chinese young adults (n* = *3,981*)							
WSSQF1	0.471	0.505	0.529	0.515	0.503	0.523	0.537
WSSQF2	0.476	0.543	0.579	0.534	0.505	0.573	0.582
PWSS	0.256	0.282	0.302	0.293	0.248	0.281	0.302

*Notes.* All *p*-values < 0.001. WSSQF1 = Weight Self-Stigma Questionnaire self-devaluation subscale; WSSQF2 = Weight Self-Stigma Questionnaire fear of enacted stigma subscale; PWSS = Perceived Weight Stigma Scale. Factor 1 = TV source; Factor 2 = Traditional media source; Factor 3 = Parent source; Factor 4 = Stranger source; Factor 5 = Social media source; Factor 6 = Significant others source; Factor 7 = Friend source

Among the Chinese adolescent subsample, the WeSEI had moderate associations with WSSQ Factor 1 (self-devaluation; range between 0.558 and 0.578) and WSSQ Factor 2 (fear of enacted stigma; range between 0.556 and 0.603). However, the WeSEI had relatively weak associations with the PWSS (range between 0.250 and 0.279) score. Among the Chinese young adult subsample, the WeSEI had small to moderate associations with WSSQ Factor 1 (self-devaluation; range between 0.471 and 0.537) and WSSQ Factor 2 (fear of enacted stigma; range between 0.476 and 0.582). However, the WeSEI had relatively small associations with PWSS (range between 0.248 and 0.302).

Table 7 shows that the higher weight group had significantly higher scores than did the non-higher weight group on Factor 2 (traditional media source; *p* = 0.004), Factor 4 (stranger source; *p* < 0.001), Factor 5 (social media source; *p* < 0.001), Factor 6 (significant others source; *p* < 0.001), and the total score (*p* = 0.02).

**Table 7:** Comparing the Weight Stigma Exposure Inventory (WeSEI) scores between higher weight and non-higher weight groups

	Mean (SD)		t (p-value)
	Non-higher weight (n = 7139)	Higher weight (n = 8852)	
Factor 1	2.14 (1.00)	2.15 (1.02)	1.00 (0.32)
Factor 2	1.98 (0.96)	2.02 (0.98)	2.86 (0.004)
Factor 3	2.17 (1.03)	2.16 (1.04)	1.06 (0.29)
Factor 4	1.77 (0.89)	1.85 (0.94)	5.39 (< 0.001)
Factor 5	1.80 (0.88)	1.86 (0.92)	3.60 (< 0.001)
Factor 6	1.73 (0.88)	1.78 (0.92)	3.72 (< 0.001)
Factor 7	2.01 (0.99)	2.01 (1.00)	0.21 (0.83)
Total score	1.94 (0.83)	1.98 (0.87)	2.42 (0.02)

*Notes*: All scores were standardized to a 1–5 scale. Factor 1 = TV source; Factor 2 = Traditional media source; Factor 3 = Parent source; Factor 4 = Stranger source; Factor 5 = Social media source; Factor 6 = Significant others source; Factor 7 = Friend source

## Discussion

The main purpose of the present study was to develop a psychometric instrument to assess exposure to weight stigma from different important sources. Initial psychometric evidence was investigated in three subsamples (i.e., Chinese adolescents, Chinese young adults, and Taiwanese young adults). More specifically, there is consistent evidence pointing to high prevalence of weight stigma experienced by adolescents and young adults due to vulnerable weight-based discrimination [10, 62, 63]. Therefore, these differing age cohorts (i.e., adolescents and young adults) were chosen participate in the present study. Moreover, previous research has shown the rising prevalence of weight stigma in China and Taiwan [10]. Therefore, examining three subsamples (i.e., Chinese adolescents, Chinese young adults, and Taiwanese young adults) may provide the potential of the WeSEI to assess exposure to weight stigma from various sources. Regarding the WeSEI score comparisons between individuals with higher weight and those without higher weight, the differences were not large. However, the significant findings indicated that individuals with higher weight may be more sensitive than those who are not higher weight in detecting observed weight stigma.

The factor structure of the WeSEI was first established using EFA and then confirmed using CFA. The results showed that the 35 WeSEI items were distributed across seven factors (five items in each factor), including sources of television, traditional media, parents, strangers, social media, significant others, and friends/peers. Additionally, the two-factor-structure of the WeSEI (interpersonal and non-interpersonal factors) was analyzed using CFA, and the results showed that the seven-factor structure had better fit than the two factor structure (the secondary analysis is presented in Supplementary Material B). Moreover, the WeSEI indicated excellent internal consistency and was supported by measurement invariance across three different subgroups (i.e., subsamples, gender, and weight status). The WeSEI total score had a small to moderate level of correlation with the WSSQ (both the self-devaluation and fear of enacted stigma subscales) and PWSS scores across different subsamples (i.e., Chinese adolescents, Chinese young adults, and Taiwanese young adults). Overall, the present study’s findings provided good preliminary psychometric evidence for the WeSEI. It is anticipated that the WeSEI will be useful in assessing exposure to key sources of weight stigma (both interpersonal and non-interpersonal sources) in various subsamples (i.e., Chinese adolescents, Chinese young adults, and Taiwanese young adults). Therefore, the WeSEI will contribute to assessing contextual factors related to weight stigma, to provide a more comprehensive and nuanced overview of individuals’ weight stigma experiences.

The present results suggested that 35 items with seven meaningful factors were selected for the WeSEI to assess exposure sources to weight stigma. The present study extended the Interpersonal Sources on Weight Stigma Scale, which assesses weight stigma across 22 domains of interpersonal sources of weight stigma [8]. The WeSEI enhances and emphasizes four main domains of interpersonal sources (i.e., parents and siblings, friends/peers, significant others, and strangers) and extends the weight stigma sources to non-interpersonal sources (i.e., social media, traditional media, television series/movies). Given the importance of weight bias from media and its harmful effects on weight bias and psychological functioning [29], the WeSEI assessed these additional sources of weight stigma to understand factors associated with weight bias in greater depth.

The present study’s results are consistent with prior studies indicating that weight stigma is most perpetuated by parents and significant others [16, 27, 37]. Even though family members usually support other family members’ health, commenting on or criticizing other members’ body weight may contribute to poor psychological health (e.g., lower body dissatisfaction, poor self-esteem, depression) [37]. Non-family sources (i.e., strangers) of stigma are also common [27]. However, weight stigma from family members has been associated with more detrimental health consequences (i.e., depression) compared to weight stigma from non-family sources [16, 27, 37]. Additionally, media sources (e.g., television, movies, social media) can be stigmatizing because they deliver health information inappropriately, promote unhealthy thin ideals, and contain explicit weight-discriminating content [16, 37]. Consequently, exposure to weight stigmatizing content through media has been associated with stronger weight-stigmatizing attitudes [37].

The present results supported the measurement invariance for the WeSEI across different subgroups (i.e., Chinese adolescents vs. Chinese young adults vs. Taiwanese young adults), gender (female vs. male), and weight status (higher weight vs. non-higher weight). A body of research exists on demographic differences in weight stigma (i.e., gender, weight status, culture) [64–66], with research suggesting that females and individuals who are higher weight face a greater risk of weight discrimination [64–66]. Notably, the present findings supporting the invariance of the WeSEI are a useful addition to this literature, by permitting the consistent interpretation of scores across individuals from diverse subsamples (e.g., gender, weight status) to assess their degree of exposure to weight stigma. Such comparisons will be useful in healthcare and research settings.

The results indicated somewhat insufficient discriminant validity between factors of the WeSEI among the participants. A strong correlation was found among television sources (Factor 1), traditional media sources (Factor 2), and social media sources (Factor 5) among the Chinese adolescent subsample. A possible explanation is that the Chinese adolescents may find it difficult to recall the precise sources of stigmatizing information. Previous research has demonstrated that adolescents have a lower performance in memory skills (i.e., recall and identification) compared to older individuals due to their still developing brains [67]. Therefore, it is likely that the present Chinese adolescent sample might not have been fully accessing their memory of exposure to weight-based stigmatizing. Another plausible explanation is that these factors (i.e., television sources, traditional sources, and social media sources) might combine into a single factor due to a strong correlation between these factors. However, additional research is needed to explore this possibility.

Moreover, there was a lack of discriminant validity for the WeSEI among parent sources (Factor 3), significant other sources (Factor 6), and friend sources (Factor 7) among Chinese adolescents and Chinese young adults. In the Chinese context, collectivist values and Confucian culture have strong influences on individual relationships (i.e., parents, friends, and significant others) [68]. Moreover, family members (i.e., parents and spouse) and friends are the most reported sources of weight stigma among adolescents and young adults [69, 70]. Therefore, it is possible that the participants in the present study considered all interpersonal factors as a whole to be a single factor from interpersonal sources. Therefore, further research is needed to establish the validity and reliability of this scale across various groups.

Although WeSEI scores were found to be significantly associated with the PWSS score, these associations were somewhat weak. Because the PWSS directly assesses experienced weight stigma (e.g., individuals personally being treated disrespectfully because of their weight), individuals who were not higher weight might not have experienced such weight stigma but still observed weight stigma from different sources assessed by the WeSEI. Given that over 40% of the present study’s participants were not classified as higher weight, it is possible that the associations between the WeSEI and PWSS were diluted by the relatively large proportion of individuals who were not higher weight.

### Implications and future directions

The present results demonstrated that all factors of the WeSEI had associations with instruments related to weight-based self-stigma and perceived weight stigma (i.e., WSSQ and PWSS). According to previous research, stigma can be considered a social construct operating due to different factors (e.g., situational, environmental, cultural, historical) with a diverse range of contexts (i.e., weight, gender) [71–73]. More specifically, when the WeSEI is used with higher-weight individuals, the associations between the seven different sources of observed stigma and health outcomes (e.g., psychological distress and low quality of life) can be investigated. Consequently, healthcare providers would know which source of observed stigma is the most critical one among higher-weight individuals in the development of poor health. For those who are not higher weight, the WeSEI could be used to examine the associations between observed stigma and their fear of becoming fat. In this regard, healthcare providers may use the WeSEI to identify those at risk for developing eating disorders/unhealthy exercise behaviors.

### Strengths and limitations

The strength of the WeSEI is its seven factors, assessing the context of weight stigma exposure, including interpersonal and non-interpersonal sources. Additionally, the scale was validated and demonstrated to have measurement invariance across various subgroups (i.e., subsamples, genders, and weight status) which enables researchers and healthcare providers to gain further precision in assessing weight stigma. The results of the WeSEI evaluation may also provide helpful guidance in planning effective interventions for the negative health outcomes of weight stigma. There are limitations in the present study. First, the study was cross-sectional using the convenience sampling method, and the samples comprised adolescents and university students, limiting generalizability to the general population. Second, the study was conducted using online self-report questions, which may have influenced the findings through increased measurement error from participants’ selection bias and social desirability. Third, the lack of test–retest reliability may limit the accuracy of the WeSEI in assessing exposure sources to weight stigma.

## Conclusions

In conclusion, the 35-item WeSEI is a valid and reliable instrument, comprising a seven-factor structure assessing exposure sources to weight stigma including interpersonal sources (i.e., parents, strangers, significant others, and friends/peers) and non-interpersonal sources (i.e., television, traditional media, and social media). Moreover, the WeSEI demonstrated equivalence as a construct across various subgroups (i.e., subsamples, gender, and weight status). Additionally, the WeSEI was positively correlated with instruments related to weight self-stigma and perceived weight stigma. Therefore, the WeSEI may be used to assess exposure sources to weight stigma across different contextual factors that might influence weight stigma. Moreover, the scale may provide preliminary evidence to identify the origins of weight stigma, which might in turn help reduce its impact. However, future research is recommended to replicate the validation and reliability of the WeSEI across different and diverse samples from other culture and countries.

## Supplementary Information

Additional file 1.

## Data Availability

The data that support the findings of the present study are available from the corresponding author upon reasonable request.
