# Supplementary material for: Assessing exposure to weight stigma: development and initial validation of the Weight Stigma Exposure Inventory (WeSEI)
Source: J Eat Disord. 2025 Jan 6;13:2. doi: 10.1186/s40337-024-01168-9 (PMC11706089; doi:10.1186/s40337-024-01168-9)
Supplement: Supplementary file 1 — Additional file 1. [file 40337_2024_1168_MOESM1_ESM.docx]

**Eating and Weight Disorders**

**Assessing Exposure to Weight Stigma: Development and Initial Validation of the Weight Stigma Exposure Inventory (WeSEI)**

Kamolthip Ruckwongpatr, I-Hua Chen, Iqbal Pramukti, Po-Ching Huang, Janet D. Latner, Kerry S. O’Brien, Xuelian Wang, Jung-Sheng Chen, Servet Üztemur, Chien-Chin Lin, Yen-Ling Chang*, Wei-Leng Chin*, Mark D. Griffiths, Chung-Ying Lin*

***Co-Correspondence:** Yen-Ling Chang; Department of Family Medicine, Cardinal Tien Hospital, New Taipei 231009, Taiwan; **E-mail:** [th.yenlingchang@gmail.com](mailto:th.yenlingchang@gmail.com);

***Co-Correspondence:** Wei-Leng. Chin; Department of Family Medicine, E-Da Hospital, Kaohsiung 824005, Taiwan; **E-mail:** [dolphin99002@gmail.com](mailto:dolphin99002@gmail.com)

***Co-Correspondence:** Chung-Ying Lin; Institute of Allied Health Sciences, College of Medicine, National Cheng Kung University, Tainan 701401, Taiwan; **E-mail:** [cylin36933@gmail.com](mailto:cylin36933@gmail.com)

**Supplementary material A**

**Procedure**

The Weight Stigma Exposure Inventor (WeSEI), a five-point Likert-type scale was generated through the following steps. First, the research team (including two occupational therapists and one physiotherapist with psychology background; one of the occupational therapists and the physiotherapist had extensive experience in weight stigma research) generated the first English version of WeSEI by focusing on two sources for experienced weight stigma (i.e., media and people). The WeSEI was created based on evidence related to stigma (social media and by individuals) (Puhl et al., 2006).

The first version of the WeSEI contained nine dimensions (i.e., online social media, traditional social media, drama, parents and sibling, friends/peers, classmates, significant others, acquaintance, and strangers) with 54 positively and negatively worded items to represent experienced weight stigma (see **Supplementary Table A1** below for details). Second, the research team consulted with two external experts working in the behavioral sciences field and had published in the academic weight stigma literature to confirm the adequate content validity of items in the WeSEI. Five researchers then discussed and evaluated removing redundant dimensions and made word changes of the items for the first version of WeSEI. Consequently, the second version of WeSEI comprised 35 items across seven dimensions (i.e., social media, traditional media, TV series/movies, parents and sibling, friends/peers, significant others, and strangers; see **Supplementary Table A2** below for details). It was then decided that the 35 items should be rated on a five-point Likert scale (1: Never; 2: Seldom; 3: Sometimes; 4: Often; 5: Almost always).

Third, the 35-item WeSEI was translated into traditional Chinese version using a standardized translation procedure (Beaton et al., 2000) of (i) forward translation (by two independent bilingual translators whose mother tongue was Chinese), (ii) back translation (by one bilingual translator whose mother tongue was Chinese but was studying in English literature with certificated English level), and (iii) expert review (by the aforementioned occupational therapist who had extensive experience in weight stigma research). After ensuring the linguistic validity of the traditional Chinese WeSEI, it was converted into simplified Chinese characters to be a simplified Chinese WeSEI. The content of the traditional Chinese WeSEI was then evaluated by six university students in Taiwan and the content of the simplified Chinese WeSEI was evaluated by 14 university students. All the university students considered that all 35 items were necessary to evaluate weight stigma exposure. Therefore, both the traditional Chinese WeSEI and simplified Chinese WeSEI were confirmed for their prefinal version for formal psychometric testing.

More specifically, exploratory factor analysis (EFA) was used for the traditional Chinese WeSEI to explore if its structure fitted with the hypothesized seven factors (i.e., seven sources), or whether some revisions are needed for its factor structure. Following this, confirmatory factor analysis was used to verify if the simplified Chinese WeSEI fitted well with the factor structure found by the EFA results for traditional Chinese WeSEI.

**References**

Beaton, D. E., Bombardier, C., Guillemin, F., & Ferraz, M. B. (2000). Guidelines for the process of cross-cultural adaptation of self-report measures. *Spine*, *25*(24), 3186-3191. <https://doi.org/10.1097/00007632-200012150-00014>

Puhl, R. M., & Brownell, K. D. (2006). Confronting and coping with weight stigma: An investigation of overweight and obese adults. *Obesity*, *14*(10), 1802-1815. <https://doi.org/10.1038/oby.2006.208>

**Supplementary material B**

**Additional results testing the Weight Stigma Exposure Inventory (WeSEI) using a second-order factor structure**

Because the WeSEI was designed according to interpersonal and non-interpersonal sources, the authors additionally examined a second-order factor structure (the four interpersonal sources [parents, strangers, significant others, and friends] embedded in second-order factor of interpersonal sources; the three non-interpersonal sources [television, traditional media, and social media] in second-order factor of non-interpersonal sources) using confirmatory factor analysis (CFA) on Chinese adolescents and Chinese young adults. The CFA fit indices were satisfactory (comparative fit index [CFI] = 0.999 and 0.992; Tucker-Lewis index [TLI] = 0.998 and 0.992; root mean square error of approximation [RMSEA] = 0.020 and 0.045; standardized root mean square residual [SRMR] = 0.030 and 0.058) but somewhat inferior to the seven-factor first-order structure (CFI = 1.000 and 0.997; TLI = 1.000 and 0.997; RMSEA = 0.010 and 0.028; SRMR = 0.018 and 0.039).

**Supplementary Table S1.** *The first version of Weight Stigma Exposure Inventor (WeSEI)*

| **From online social media** |
| --- |
| 1. I have learned negative descriptions about weight (e.g., poorer image body) from online social media (e.g., Facebook, websites, and blogs). 2. I have learned negative features about people who are overweight (e.g., fat people are useless and lazy) from online social media (e.g., Facebook, websites, and blogs). 3. I have learned that being slim can be more attractive from online social media (e.g., Facebook, websites, and blogs). 4. I have seen negative emotions towards people who are overweight (e.g., I hate fat people) from online social media (e.g., Facebook, websites, and blogs). 5. I have seen internet memes and jokes about weight (e.g., fat people are unattractive, unfriendly, unworthy) from online social media (e.g., Facebook, websites, and blogs). 6. I have seen negative behavior descriptions towards people who are overweight (e.g., I kick those with a fat ass) from online social media (e.g., Facebook, websites, and blogs). |
| **From traditional media** |
| 1. I have learned negative descriptions about weight (e.g., poorer image body) from traditional media (e.g., television, newspapers, and magazines). 2. I have learned negative features about people who are overweight (e.g., fat people are useless and lazy) from traditional media (e.g., television, newspaper, and magazines). 3. I have learned that being slim can be more attractive from traditional media (e.g., television, newspaper, and magazines). 4. I have seen negative emotions towards people who are overweight (e.g., I hate fat people) from traditional media (e.g., television, newspaper, and magazines). 5. I have seen jokes about weight (e.g., fat people are unattractive, unfriendly, unworthy) from traditional media (e.g., television, newspaper, and magazines). 6. I have seen negative behaviors towards people who are overweight (e.g., I kick those with a fat ass) from traditional media (e.g., television, newspaper, and magazines). |
| **From drama** |
| 1. The dramas or movies I have watched describe weight in a negative manner (e.g., poorer image body). 2. The dramas or movies I have watched portray people who are overweight as having negative features (e.g., they are useless and lazy). 3. The dramas or movies I have watched portray people with a slim shape as being attractive. 4. The dramas or movies I have watched showed that people have negative emotions towards being overweight (e.g., dislike or disgust) . 5. The dramas or movies I have watched showed that people who are overweight are teased. 6. The dramas or movies I have watched showed that people who are overweight experiencing negative behaviors (e.g., being bullied). |
| **From parents and/or siblings** |
| 1. I have heard my parents and/or siblings describe weight negatively (e.g., poorer image body). 2. I have heard my parents and/or siblings portray people who are overweight negatively (e.g., fat people are useless and lazy). 3. I have heard my parents and/or siblings portray people with a slim shape as being attractive. 4. I have heard my parents and/or siblings express negative emotions towards being overweight (e.g., I hate fat people). 5. I have heard my parents and/or siblings joking or teasing about weight (e.g., fat people are unattractive, unfriendly, unworthy). 6. I have seen my parents and/or siblings use negative behaviors towards people who are overweight (e.g., bullying). |
| **From friends/peers** |
| 1. I have heard my friends/peers describe weight negatively (e.g., poorer image body). 2. I have heard my friends/peers portray people who are overweight negatively (e.g., fat people are useless and lazy). 3. I have heard my friends/peers portray people with a slim shape as being attractive. 4. I have heard my friends/peers express negative emotions towards being overweight (e.g., I hate fat people). 5. I have heard my friends/peers joking or teasing about weight (e.g., fat people are unattractive, unfriendly, unworthy). 6. I have seen my friends/peers use negative behaviors towards people who are overweight (e.g., bullying). |
| **From classmates** |
| 1. I have heard my classmates describe weight negatively (e.g., poorer image body). 2. I have heard my classmates portray people who are overweight negatively (e.g., fat people are useless and lazy). 3. I have heard my classmates portray people with a slim shape as being attractive. 4. I have heard my classmates express negative emotions towards being overweight (e.g., I hate fat people). 5. I have heard my classmates joking or teasing about weight (e.g., fat people are unattractive, unfriendly, unworthy). 6. I have seen my classmates use negative behaviors towards people who are overweight (e.g., bullying). |
| **From significant others** |
| 1. I have heard my significant others describe weight negatively (e.g., poorer image body). 2. I have heard my significant others portray people who are overweight negatively (e.g., fat people are useless and lazy). 3. I have heard my significant others portray people with a slim shape as being attractive. 4. I have heard my significant others express negative emotions towards being overweight (e.g., I hate fat people). 5. I have heard my significant others joking or teasing about weight (e.g., fat people are unattractive, unfriendly, unworthy). 6. I have seen my significant others use negative behaviors towards people who are overweight (e.g., bullying). |
| **From acquaintances** |
| 1. I have heard my acquaintances describe weight negatively (e.g., poorer image body). 2. I have heard my acquaintances portray people who are overweight negatively (e.g., fat people are useless and lazy). 3. I have heard my acquaintances portray people with a slim shape as being attractive. 4. I have heard my acquaintances express negative emotions towards being overweight (e.g., I hate fat people). 5. I have heard my acquaintances joking or teasing about weight (e.g., fat people are unattractive, unfriendly, unworthy). 6. I have seen my acquaintances use negative behaviors towards people who are overweight (e.g., bullying). |
| **From strangers** |
| 1. I have heard strangers describe weight negatively (e.g., poorer image body). 2. I have heard strangers portray people who are overweight negatively (e.g., fat people are useless and lazy). 3. I have heard strangers about people with a slim shape as being attractive. 4. I have heard strangers expressing negative emotions towards being overweight (e.g., I hate fat people). 5. I have heard strangers joking or teasing about weight (e.g., fat people are unattractive, unfriendly, unworthy). 6. I have seen strangers engaging in negative behaviors towards people who are overweight (e.g., bullying). |

**Supplementary Table S2.** *The second version of Weight Stigma Exposure Inventor (WeSEI) and its Chinese versions*

**Instruction:** **Please indicate how often you have experienced the following in the past 12 months.**

| **From social media (e.g., Facebook, websites, and blogs)** | **Never** | **Seldom** | **Sometimes** | **Often** | **Almost always** |
| --- | --- | --- | --- | --- | --- |
| 1. I have observed negative statements about weight on social media (e.g., overweight people are pathetic; fat people are useless and lazy). |  |  |  |  |  |
| 1. I have observed that people consider being slim is more attractive than being overweight on social media. |  |  |  |  |  |
| 1. I have observed that people don’t like individuals who are overweight on social media. |  |  |  |  |  |
| 1. I have observed internet memes and jokes about weight on social media (e.g., fat people are unattractive or unfriendly). |  |  |  |  |  |
| 1. I have observed people writing down their unfriendly behaviors toward people who are overweight on social media (e.g., teasing a fat person). |  |  |  |  |  |
| **From traditional media (e.g., newspaper, magazines, and books)** | **Never** | **Seldom** | **Sometimes** | **Often** | **Almost always** |
| 1. I have observed negative statements about weight in traditional media (e.g., overweight people are pathetic; fat people are useless and lazy). |  |  |  |  |  |
| 1. I have observed that people consider being slim is more attractive than being overweight in traditional media. |  |  |  |  |  |
| 1. I have observed that people don’t like individuals who are overweight in traditional media. |  |  |  |  |  |
| 1. I have observed jokes about weight in traditional media (e.g., fat people are unattractive or unfriendly). |  |  |  |  |  |
| 1. I have observed people writing down their unfriendly behaviors toward people who are overweight in traditional media (e.g., teasing a fat person). |  |  |  |  |  |
| **From TV Series/ Movies** | **Never** | **Seldom** | **Sometimes** | **Often** | **Almost always** |
| 1. The TV series/movies I watched portrayed individuals who are overweight in a negative way (e.g., overweight people are pathetic; fat people are useless and lazy). |  |  |  |  |  |
| 1. The TV series/movies I watched portrayed people with a slim shape as being attractive. |  |  |  |  |  |
| 1. The TV series/movies I watched typically suggested that TV characters don’t like people who are overweight. |  |  |  |  |  |
| 1. The TV series/movies I watched showed that people who are overweight were teased. |  |  |  |  |  |
| 1. The TV series/movies I watched showed that people who are overweight were treated badly (e.g., teased or bullied) |  |  |  |  |  |
| **From parents and siblings** | **Never** | **Seldom** | **Sometimes** | **Often** | **Almost always** |
| 1. My parents and siblings have negative beliefs about weight (e.g., overweight people are pathetic; fat people are useless and lazy). |  |  |  |  |  |
| 1. My parents and siblings think that people with a slim shape are more attractive than those who are overweight. |  |  |  |  |  |
| 1. My parents and siblings don’t like people who are overweight. |  |  |  |  |  |
| 1. My parents and siblings make jokes or tease people about their weight (e.g., fat people are unattractive or unfriendly). |  |  |  |  |  |
| 1. My parents and siblings have treated people who are overweight badly (e.g., teasing or bullying). |  |  |  |  |  |
| **From friends/peers** | **Never** | **Seldom** | **Sometimes** | **Often** | **Almost always** |
| 1. My friends/peers have negative beliefs about weight (e.g., overweight people are pathetic; fat people are useless and lazy). |  |  |  |  |  |
| 1. My friends/peers think/believe that people with a slim shape are more attractive than those who are overweight. |  |  |  |  |  |
| 1. My friends/peers don’t like people who are overweight |  |  |  |  |  |
| 1. My friends/peers make jokes or tease people about their weight (e.g., fat people are unattractive, unfriendly, unworthy). |  |  |  |  |  |
| 1. My friends/peers have treated people who are overweight badly (e.g., teasing or bullying). |  |  |  |  |  |
| **From a significant other (e.g., partner/wife/boyfriend)** | **Never** | **Seldom** | **Sometimes** | **Often** | **Almost always** |
| 1. My significant other has negative beliefs about weight (e.g., overweight people are pathetic; fat people are useless and lazy). |  |  |  |  |  |
| 1. My significant other finds people with a slim shape more attractive than those who are overweight. |  |  |  |  |  |
| 1. My significant other doesn’t like people who are overweight. |  |  |  |  |  |
| 1. My significant other makes jokes or teases people about their weight (e.g., fat people are unattractive, unfriendly). |  |  |  |  |  |
| 1. My significant other treats people who are overweight badly (e.g., teasing or bullying). |  |  |  |  |  |
| **From strangers** | **Never** | **Seldom** | **Sometimes** | **Often** | **Almost always** |
| 1. I have observed negative beliefs about weight (e.g., overweight people are pathetic; fat people are useless and lazy) from strangers. |  |  |  |  |  |
| 1. I have observed that strangers find people with a slim shape more attractive than those who are overweight. |  |  |  |  |  |
| 1. I have observed that strangers don’t like people who are overweight. |  |  |  |  |  |
| 1. I have seen strangers making jokes or tease people about their weight (e.g., fat people are unattractive or unfriendly). |  |  |  |  |  |
| 1. I have seen strangers treating people who are overweight badly (e.g., teasing or bullying). |  |  |  |  |  |

Chinese version using Traditional Chinese characters

| **說明：請指出在過去12個月中，您同意以下敘述的頻率** | 從未 | 很少 | 有時 | 時常 | 幾乎每天使用 |
| --- | --- | --- | --- | --- | --- |
| **在社群媒體使用上（例如：臉書、網頁和部落格）** | | | | | |
| 1.我曾在社群媒體上看到關於體重的負面敘述（例如：過重是很可悲的；胖子都沒用且懶惰）。 |  |  |  |  |  |
| 2.我曾在社群媒體上看到人們認為苗條的人比過重/肥胖的人更有魅力 |  |  |  |  |  |
| 3.我曾在社群媒體上看到人們評論不喜歡過重/肥胖的人 |  |  |  |  |  |
| 4.我曾在社群媒體上看到關於體重的網路惡搞與笑話（例如：胖子都不好看或不友善） |  |  |  |  |  |
| 5.我曾在社群媒體上看到人們寫下他們針對過重/肥胖的人的不友善行為（例如：我取笑了一個胖子） |  |  |  |  |  |
| **在傳統媒體上（例如：電視、報紙、雜誌和書籍）** | | | | | |
| 6.我曾在傳統媒體上看到關於體重的負面敘述（例如：過重是很可悲的；胖子都沒用且懶惰） |  |  |  |  |  |
| 7.我曾在傳統媒體上看到人們認為苗條的人比過重/肥胖的人更有魅力 |  |  |  |  |  |
| 8.我曾在傳統媒體上看到人們評論不喜歡過重/肥胖的人 |  |  |  |  |  |
| 9.我曾在傳統媒體上看到關於體重的笑話（例如：胖子都不好看或不友善） |  |  |  |  |  |
| 10.我曾在傳統媒體上看過人們寫下他們針對過重/肥胖的人的不友善行為（例如：我取笑了一個胖子） |  |  |  |  |  |
| **在電視劇/電影上** | | | | | |
| 11.我看過的電視劇/電影用負面的方式來描繪過重/肥胖的人（例如：過重是很可悲的；胖子都沒用且懶惰） |  |  |  |  |  |
| 12.我看過的電視劇/電影描繪身形苗條的人是有魅力的 |  |  |  |  |  |
| 13.我看過的電視劇/電影通常暗示電視角色不喜歡過重/肥胖的人 |  |  |  |  |  |
| 14.我看過的電視劇/電影演出過重/肥胖的人是被取笑的 |  |  |  |  |  |
| 15.我看過的電視劇/電影演出過重/肥胖的人被惡劣地對待（例如：被取笑或被霸凌） |  |  |  |  |  |
| **從父母與兄弟姊妹** | | | | | |
| 16.我的父母與兄弟姊妹對體重有負面的看法（例如：過重很可悲；胖子都沒用且懶惰） |  |  |  |  |  |
| 17.我的父母與兄弟姊妹認為身形苗條的人比那些過重/肥胖的人更有魅力 |  |  |  |  |  |
| 18.我的父母與兄弟姊妹不喜歡過重/肥胖的人 |  |  |  |  |  |
| 19.我的父母與兄弟姊妹會嘲笑或開體重的玩笑（例如：胖子都不好看或不友善） |  |  |  |  |  |
| 20.我的父母與兄弟姊妹曾惡劣地對待過重/肥胖的人（例如：嘲笑或霸凌） |  |  |  |  |  |
| **從朋友/同儕** | | | | | |
| **我快做完了，我很專心。此題請回答「幾乎每天使用」** |  |  |  |  |  |
| 21.我的朋友/同儕對體重有負面的看法（例如：過重是很可悲的；胖子都沒用且懶惰） |  |  |  |  |  |
| 22.我的朋友/同儕認為/相信身形苗條的人比過重/肥胖的人更有魅力 |  |  |  |  |  |
| 23.我的朋友/同儕不喜歡過重/肥胖的人 |  |  |  |  |  |
| 24.我的朋友/同儕會嘲笑或開體重的玩笑（例如：胖子都不好看、不友善、無法融入群體） |  |  |  |  |  |
| 25.我的朋友/同儕曾惡劣地對待過重/肥胖的人（例如：嘲笑或霸凌） |  |  |  |  |  |
| **從我身邊重要的人（例如：伴侶/老婆/男友）** | | | | | |
| 26.我身邊重要的人對體重有負面的看法（例如：過重是很可悲的；胖子都沒用且懶惰） |  |  |  |  |  |
| 27.我身邊重要的人認為身形苗條的人比過重/肥胖的人更有魅力 |  |  |  |  |  |
| 28.我身邊重要的人不喜歡過重/肥胖的人 |  |  |  |  |  |
| 29.我身邊重要的人會嘲笑或開體重的玩笑（例如：胖子都不好看、不友善） |  |  |  |  |  |
| 30.我身邊重要的人曾惡劣地對待過重/肥胖的人（例如：嘲笑或霸凌） |  |  |  |  |  |
| **從陌生人** | | | | | |
| 31.我曾觀察到陌生人對體重的負面看法（例如：過重是很可悲的；胖子都沒用且懶惰） |  |  |  |  |  |
| 32.我曾觀察到陌生人認為身形苗條的人比過重/肥胖的人更有魅力 |  |  |  |  |  |
| 33.我曾觀察到陌生人不喜歡過重/肥胖的人 |  |  |  |  |  |
| 34.我曾發現陌生人會嘲笑或開體重的玩笑（例如：胖子都不好看或不友善） |  |  |  |  |  |
| 35.我曾發現陌生人惡劣地對待過重/肥胖的人（例如：嘲笑或霸凌） |  |  |  |  |  |

Chinese version using Simplified Chinese characters

**说明：请指出在过去12个月中，您同意以下叙述的频率**

| **在社交媒体上（例如：微博、网页和博客）** | **从未** | **很少** | **有时** | **时常** | **几乎总是** |
| --- | --- | --- | --- | --- | --- |
| 1. 我曾在社交媒体上看到关于体重的负面报导（例如：超重是很可悲   的；胖子都没用且懒惰）。 |  |  |  |  |  |
| 2. 我曾在社交媒体上看到人们认为苗条的人比超重/肥胖的人更有魅力。 |  |  |  |  |  |
| 3. 我曾在社交媒体上看到人们不喜欢超重/肥胖的人。 |  |  |  |  |  |
| 4. 我曾在社交媒体上看到关于体重的网络流行梗与笑话（例如：胖子都  不好看或不友善）。 |  |  |  |  |  |
| 5. 我曾在社交媒体上看到人们写下他们针对超重/肥胖的人的不友善行为  （例如：我取笑了一个胖子）。 |  |  |  |  |  |
| **在传统媒体上（例如：电视、报纸、杂志和书籍）** | **从未** | **很少** | **有时** | **时常** | **几乎总是** |
| 6. 我曾在传统媒体上看到关于体重的负面报导（例如：超重是很可悲  的；胖子都没用且懒惰）。 |  |  |  |  |  |
| 7. 我曾在传统媒体上看到人们认为苗条的人比超重/肥胖的人更有魅力。 |  |  |  |  |  |
| 8. 我曾在传统媒体上看到人们不喜欢超重/肥胖的人。 |  |  |  |  |  |
| 9. 我曾在传统媒体上看到关于体重的笑话（例如：胖子都不好看或不友  善）。 |  |  |  |  |  |
| 10.我曾在传统媒体上看过人们写下他们针对超重/肥胖的人的不友善行  为（例如：我取笑了一个胖子）。 |  |  |  |  |  |
| **在电视剧/电影上** | **从未** | **很少** | **有时** | **时常** | **几乎总是** |
| 11.我看过的电视剧/电影用负面的方式来描绘超重/肥胖的人（例如：过  重是很可悲的；胖子都没用且懒惰）。 |  |  |  |  |  |
| 12.我看过的电视剧/电影描绘身材苗条的人是有魅力的。 |  |  |  |  |  |
| 13.我看过的电视剧/电影通常暗示电视角色不喜欢超重/肥胖的人。 |  |  |  |  |  |
| 14.我看过的电视剧/电影中，超重/肥胖的人是会被取笑的。 |  |  |  |  |  |
| 15.我看过的电视剧/电影中，超重/肥胖的人被恶劣地对待（例如：被取  笑或被霸凌）。 |  |  |  |  |  |
| **从父母与兄弟姐妹** | **从未** | **很少** | **有时** | **时常** | **几乎总是** |
| 16.我的父母与兄弟姐妹对体重有负面的看法（例如：超重很可悲；胖子  都没用且懒惰）。 |  |  |  |  |  |
| 17.我的父母与兄弟姐妹认为身材苗条的人比那些超重/肥胖的人更有魅  力。 |  |  |  |  |  |
| 18.我的父母与兄弟姐妹不喜欢超重/肥胖的人。 |  |  |  |  |  |
| 19.我的父母与兄弟姐妹会嘲笑或开体重的玩笑（例如：胖子都不好看或  不友善）。 |  |  |  |  |  |
| 20.我的父母与兄弟姐妹曾恶劣地对待超重/肥胖的人（例如：嘲笑或霸  凌）。 |  |  |  |  |  |
| **从朋友/同伴** | **从未** | **很少** | **有时** | **时常** | **几乎总是** |
| 21.我的朋友/同伴对体重有负面的看法（例如：超重是很可悲的；胖子  都没用且懒惰）。 |  |  |  |  |  |
| 22.我的朋友/同伴认为/相信身材苗条的人比超重/肥胖的人更有魅力。 |  |  |  |  |  |
| 23.我的朋友/同伴不喜欢超重/肥胖的人。 |  |  |  |  |  |
| 24.我的朋友/同伴会嘲笑或开体重的玩笑（例如：胖子都不好看、不友  善、无法融入群体）。 |  |  |  |  |  |
| 25.我的朋友/同伴曾恶劣地对待超重/肥胖的人（例如：嘲笑或霸凌）。 |  |  |  |  |  |
| **从重要他人（例如：伴侣/妻子/丈夫/女友/男友）** | **从未** | **很少** | **有时** | **时常** | **几乎总是** |
| 26.我的重要他人对体重有负面的看法（例如：超重是很可悲的；胖子都  没用且懒惰）。 |  |  |  |  |  |
| 27.我的重要他人认为身材苗条的人比超重/肥胖的人更有魅力。 |  |  |  |  |  |
| 28.我的重要他人不喜欢超重/肥胖的人。 |  |  |  |  |  |
| 29.我的重要他人会嘲笑或开体重的玩笑（例如：胖子都不好看、不友  善）。 |  |  |  |  |  |
| 30.我的重要他人曾恶劣地对待超重/肥胖的人（例如：嘲笑或霸凌）。 |  |  |  |  |  |
| **从陌生人** | **从未** | **很少** | **有时** | **时常** | **几乎总是** |
| 31.我曾观察到陌生人对体重的负面看法（例如：超重是很可悲的；胖子  都没用且懒惰）。 |  |  |  |  |  |
| 32.我曾观察到陌生人认为身材苗条的人比超重/肥胖的人更有魅力。 |  |  |  |  |  |
| 33.我曾观察到陌生人不喜欢超重/肥胖的人。 |  |  |  |  |  |
| 34.我曾发现陌生人会嘲笑或开体重的玩笑（例如：胖子都不好看或不友  善）。 |  |  |  |  |  |
| 35.我曾发现陌生人恶劣地对待超重/肥胖的人（例如：嘲笑或霸凌）。 |  |  |  |  |  |

**Supplementary Table S3**. Mardia’s test of multivariate normality

|  | Taiwanese young adult sample  (n=887) | Chinese adolescent sample  (n=11,123) | Chinese young adult sample  (n=3981) |
| --- | --- | --- | --- |
| Multivariate skewness statistic | 24976.883 | 713400.256 | 237027.475 |
| *p*-value of skewness | <0.001 | <0.001 | <0.001 |
| Multivariate kurtosis statistic | 144.090 | 4983.451 | 1660.650 |
| *p*-value of kurtosis | <0.001 | <0.001 | <0.001 |

**Supplementary Table S4**. Inter-factor correlations among the factors extracted from the exploratory factor analysis using Taiwanese young adult sample (n = 887)

|  | F1 | F2 | F3 | F4 | F5 | F6 | F7 | F8 |
| --- | --- | --- | --- | --- | --- | --- | --- | --- |
| F1 | (0.939/0.940) |  |  |  |  |  |  |  |
| F2 | 0.658 | (0.936/0.936) |  |  |  |  |  |  |
| F3 | 0.425 | 0.418 | (0.907/0.913) |  |  |  |  |  |
| F4 | 0.607 | 0.515 | 0.404 | (0.926/0.928) |  |  |  |  |
| F5 | 0.680 | 0.698 | 0.441 | 0.540 | (0.910/0.911) |  |  |  |
| F6 | 0.312 | 0.371 | 0.593 | 0.402 | 0.359 | (0.898/0.904) |  |  |
| F7 | 0.243 | 0.385 | 0.427 | 0.334 | 0.308 | 0.553 | (0.891/0.894) |  |
| F8 | 0.186 | -0.041 | 0.045 | 0.101 | 0.112 | 0.064 | -0.210 | (--/--) |

F1 = TV source; F2 = Traditional media source; F3 = Parent source; F4 = Stranger source; F5 = Social media source; F6 = Significant others source; F7 = Friend source; F8 was not used because it does not have items with strong factor loadings. Values in the diagonal line within parentheses are Cronbach’s α/McDonald’s ω.

**Supplementary Table S5.** Inter-factor correlations among the factors estimated in the confirmatory factor analysis using Chinese adolescent sample (n = 11,123)

|  | F1 | F2 | F3 | F4 | F5 | F6 | F7 |
| --- | --- | --- | --- | --- | --- | --- | --- |
| F1 | (0.971/0.971) |  |  |  |  |  |  |
| F2 | 0.860 | (0.974/0.974) |  |  |  |  |  |
| F3 | 0.763 | 0.782 | (0.969/0.970) |  |  |  |  |
| F4 | 0.804 | 0.774 | 0.790 | (0.978/0.978) |  |  |  |
| F5 | 0.849 | 0.885 | 0.720 | 0.770 | (0.955/0.955) |  |  |
| F6 | 0.730 | 0.763 | 0.893 | 0.816 | 0.694 | (0.980/0.980) |  |
| F7 | 0.773 | 0.789 | 0.907 | 0.845 | 0.737 | 0.922 | (0.973/0.973) |

F1 = TV source; F2 = Traditional media source; F3 = Parent source; F4 = Stranger source; F5 = Social media source; F6 = Significant others source; F7 = Friend source. Values in the diagonal line within parentheses are Cronbach’s α/McDonald’s ω.

**Supplementary Table S6.** Inter-factor correlations among the factors estimated in the confirmatory factor analysis using Chinese young adult sample (n = 3981)

|  | F1 | F2 | F3 | F4 | F5 | F6 | F7 |
| --- | --- | --- | --- | --- | --- | --- | --- |
| F1 | (0.966/0.967) |  |  |  |  |  |  |
| F2 | 0.736 | (0.971/0.971) |  |  |  |  |  |
| F3 | 0.642 | 0.737 | (0.957/0.957) |  |  |  |  |
| F4 | 0.766 | 0.686 | 0.704 | (0.968/0.968) |  |  |  |
| F5 | 0.791 | 0.837 | 0.663 | 0.733 | (0.938/0.938) |  |  |
| F6 | 0.579 | 0.734 | 0.842 | 0.699 | 0.643 | (0.968/0.968) |  |
| F7 | 0.639 | 0.738 | 0.879 | 0.743 | 0.688 | 0.922 | (0.953/0.953) |

F1 = TV source; F2 = Traditional media source; F3 = Parent source; F4 = Stranger source; F5 = Social media source; F6 = Significant others source; F7 = Friend source. Values in the diagonal line within parentheses are Cronbach’s α/McDonald’s ω.
